# A tool for early estimation of influenza vaccination coverage in Spanish general population and healthcare workers in the 2018–19 season: the Gripómetro

**DOI:** 10.1186/s12889-022-13193-x

**Published:** 2022-04-25

**Authors:** Javier Díez-Domingo, Esther Redondo Margüello, Raúl Ortiz de Lejarazu Leonardo, Ángel Gil de Miguel, José María Guillén Ortega, Jesús Rincón Mora, Federico Martinón-Torres

**Affiliations:** 1 Fundación para el Fomento de la Investigación Sanitaria y Biomédica de la Comunitat Valenciana (FISABIO), 46020 Valencia, Spain; 2 Centro de Salud Internacional Madrid Salud, Ayuntamiento de Madrid, Madrid, Spain; 3 Centro Nacional de Gripe de Valladolid, Valladolid, Spain; 4 Departamento de Especialidades Médicas y Salud Pública, Universidad Rey Juan Carlos, Madrid, Spain; 5 Sanofi España, Madrid, Spain; 6 Instituto Análisis e Investigación, Madrid, Spain; 7 Servicio de Pediatría, Hospital Clínico Universitario de Santiago, Santiago de Compostela, Spain; 8 Grupo de Genética, Infecciones y Vacunas en Pediatría (GENVIP), Instituto de Investigación Sanitaria de Santiago, Santiago de Compostela, Spain

**Keywords:** Influenza, Influenza vaccine, Vaccine coverage, Surveillance

## Abstract

**Background:**

Electronic vaccine registries are not yet widely established. There is a need to real-time monitor influenza vaccine coverage, which may raise awareness to risk groups and professionals, and eventually allow to adopt tailored measures during the vaccination campaign. To evaluate the utility of the “Gripómetro”, a demographic study designed to monitor national and regional influenza vaccine coverage on a weekly basis in Spain.

**Methods:**

Quantitative study based on surveys of the Spanish population between 18–80 years and a sample of primary care doctors and nurses randomly selected. Pre-proportional fixation has been established by Autonomous Communities and age group to guarantee the representativeness of all the autonomies.

**Results:**

Interviews were conducted in 3400 households of general population and 807 respondents among health care professionals. We found that the results of influenza vaccination coverage in the population ≥ 65 years obtained by the Gripómetro for 2018–2019 season were mostly comparable with the official data presented by the Ministry of Health after the end of the vaccination campaign.

**Conclusions:**

The Gripómetro is a robust research method that provides real-time data and trends for influenza vaccine coverage along with other useful information related to vaccination such as intention to vaccinate, motivation and barriers to vaccination.

**Supplementary Information:**

The online version contains supplementary material available at 10.1186/s12889-022-13193-x.

## Introduction

Influenza is a public health issue due to its impact on society and the health system, as it is an infectious disease that affects people of all ages and a significant proportion of the population. It can lead to serious clinical symptoms and even death, especially in certain risk groups, such as children under 2 years old, adults ≥ 65 years old, pregnant women and patients with chronic diseases [1–3]. According to the World Health Organization (WHO), influenza epidemics are estimated to affect 5%-15% of the population annually, causing between 3 and 5 million cases of serious illness and 290 000 to 650 000 deaths a year globally [1]. In Spain, the mean mortality rate associated with influenza is estimated to be between 1.61 and 3.37 deaths per 100 000 population per year, with the highest concentration of cases occurring in people with risk factors [4, 5].

Influenza vaccination is the most effective way of preventing influenza virus infection and its complications [6, 7]. The European Commission and the Spanish Ministry of Health, Consumer Affairs and Social Welfare recommend the establishment or strengthening of strategies aimed at improving vaccination coverage in the 2019–20 season in risk groups. The objectives for the 2020–21 season in Spain are to achieve or exceed 75% vaccination coverage in adults ≥ 65 years old and in healthcare workers, and to exceed 60% in pregnant women [8]. Nonetheless, in the 2016–17 season, the median vaccination coverage rate in 19 countries of the European Union (EU), including Spain, was estimated at 47.1% (coverage range of analysed countries 2.0%–72.8%) in adults ≥ 65 years and 30.2% (coverage range of analysed countries 15.6%–63.2%) in healthcare workers [9]. In Spain, in the 2018–19 season, the overall vaccination coverage was 54.3% (range 41.5%–64.5%) in individuals ≥ 65 years (with a 9-point drop after the 2009–2010 season), 33.9% (range 21.0%–58.7%) among healthcare workers, and 38.5% (range 16.1%–54.5%) in pregnant women [10].

However, in many European countries, vaccination coverage in risk groups is not properly monitored. According to data from the European Centre for Disease Prevention and Control (ECDC) for the 2016–17 season, only 19 of the 30 participating Member states provided data on vaccination coverage in older adults, 12 in healthcare workers, 9 in pregnant women, 7 in patients with chronic conditions and 5 in residents of nursing homes and long-term care facilities (Spain did not provide data for the latter 3 groups) [9].

Estimating vaccine coverage in real time allows continuous evaluation of vaccine use, and is essential to assess its impact on the population, both in terms of effectiveness and safety. Countries such as Finland, Norway, Denmark and the Netherlands have national vaccination registries in which data are electronically transferred from electronic patient health records, allowing continuous surveillance [11, 12]. Some Spanish Autonomous Regions have already similar systems, as the Nominal Vaccine Registry (RVN) of the Conselleria de Sanitat Universal i Salut Pública de la Comunidad Valenciana in which the administered vaccines in both public and private vaccination points are declared from the electronic medical records [13]. However, there is no standardized national system allowing real time monitorization of vaccination coverage in Spain. Instead, national influenza coverage data is collected by the Ministry of Health from the different Autonomous Communities and released at end of each season.

The aim of this paper is to present the Gripometro, a demographic study which, accompanied by a website, allows vaccination coverage in Spain to be monitored, and provides verified data on the evolution of the vaccination campaign in real time [14]. This tool enables us to assess the impact of these campaigns, identify gaps in coverage and analyse their time trends, allowing us to inform and guide health systems and the population about of the vaccination campaign, and to correct or emphasize the importance and need for influenza vaccination in risk groups.

## Methods

This study targets 2 population groups: (i) on the one hand, the general population residing in Spain, with a particular interest in data on children < 5 years of age, adults ≥ 65 years of age and individuals included in other risk groups, and (ii) on the other, healthcare workers in the field of primary care, a risk group that, as mentioned above, is not only exposed to an increased risk of influenza virus infection, but is an important vector of transmission for patients.

### Research in the general population and healthcare workers

Research in the general population was based on a national quantitative study using computer-assisted telephone interviewing (CATI) via landlines to individuals randomly selected from a population aged between 18 and 80 years resident in Spain. Individuals were asked to answer a semi-structured questionnaire with an average duration of 15 min. This questionnaire collected data to estimate the vaccination coverage of both the individual (respondent) and all members of the household. It also questioned the respondent on sociodemographic and attitudinal variables with regard to influenza and vaccination (Supplementary material S1).

For healthcare workers, a national quantitative study was conducted through an online survey of a randomly selected panel of doctors and nurses who practiced in public primary care facilities (Supplementary material S2). In this case, the survey was followed up by a telephone call.

To analyse the trend in vaccination coverage, the survey — both in the general population and in healthcare workers — was conducted in weekly waves (Monday to Friday) over 6 consecutive weeks, starting 2 weeks after commencement of the vaccination campaign in each autonomous region (first wave from November 5th to 11th, second wave from November 12th to 18th, third wave from November 19th to 25th, fourth wave from November 26th to December 2nd, fifth wave from December 3rd to 9th and sixth wave from December 10th to 16th). Results estimates are made per week.

In order to obtain more specific data, both for sampling and analysis, the general population was divided into 2 large groups based on age: (i) adults aged 18 to 64 years and (ii) adults aged 65 to 80 years. In this way, sufficiently representative coverage data could be obtained, according to population criteria, assuming a margin of error of ± 4.4% for children under 18 years, ± 1.7% for the population aged 18 to 64 years, and ± 1.5% for the population aged ≥ 65 years (errors calculated for an infinite universe and a 95.5% confidence level in the assumption of maximum indeterminacy *p* = q = 0.5). A total of 807 surveys were carried out in healthcare workers, a number that also obtained sufficiently representative coverage data for the group, assuming a margin of error of ± 3.5% (error calculated for an infinite universe and a 95.5% confidence level in the assumption of maximum indeterminacy *p* = q = 0.5).

Although the scope of the study was national, in order to ensure that the results were representative of all regions and comparable to each other, fixed quotas were established by autonomous region and by age group. Each region was assigned 200 questionnaires in the general population: 50 in the group aged 18 to 64 years and 150 in the group aged 65 to 80 years. Only the autonomous cities of Ceuta and Melilla were excluded from the study. In the case of healthcare workers, 15 questionnaires were assigned to each autonomous region, distributing the remaining questionnaires in proportion to the actual weight of the doctors and nurses in each region. To return proportionality to the sample and thus balance any possible mismatches resulting from the fixed quota per region, the results were weighted on the basis of the population universe for autonomous regions and sex and age group for the general population, or on the basis of the national universe of primary care physicians and nurses in each autonomous region for healthcare workers.

The results of vaccination coverage in the general population were based on the answers given by the respondents to the following questions: (i) Starting with you and then the other members of your household, have you already been vaccinated, or are you planning to be vaccinated against the flu during this 2018–2019 campaign? (ii) Indicate your sex; (iii) Indicate your age. In the case of healthcare workers, the results were based on the questions: (i) Have you already been vaccinated against the flu in this 2018–2019 campaign? (ii) What are your main reasons for deciding to get vaccinated?

## Results

### Analysis of general population coverage

#### Data by age groups

**Fig. 1:**
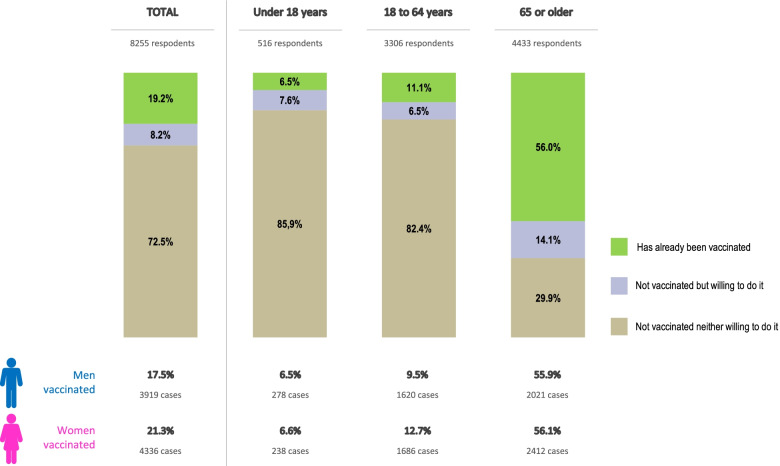
Analysis of influenza vaccination coverage in the overall Spanish population participating in the interviews (2018/2019)

In total, interviews were conducted in 3400 households: 850 among the population aged 18 to 64, and 2550 among the population aged 65 to 80. With an estimated mean of 2.43 persons per household, data were obtained for 8255 individuals (3919 men and 4336 women), of whom 19.2% had been vaccinated against influenza (17.1% of men and 21.3% of women). By age subgroup, (i) among children under 18 years of age (*n* = 516, 278 boys and 238 girls), 6.5% had been vaccinated, with no differences between sexes; (ii) in the 18 to 64 age group (*n* = 3306, 1620 men and 1686 women), 11.1% had been vaccinated, 9.5% of men and 12.7% of women; and (iii) among adults ≥ 65 years (*n* = 4433, 2021 men and 2412 women), more than half had been vaccinated (56.0%), 55.9% of men and 56.1% of women (Fig. 1).

In people under 18 years of age (*n* = 516; 278 boys and 238 girls), individuals investigated reported that 6.5% (33 patients) had been vaccinated against influenza, with no differences between sexes (Table 1). Two-thirds of these patients had no chronic conditions and none were pregnant women. By age subgroups, among children aged 0 to 5 years (*n* = 74, small sample base), the vast majority (90.5%) had not been vaccinated nor did they intend to do so; 6.4% of children aged 5 to 14 years (*n* = 294) had been vaccinated, while vaccination coverage was 7.4% among children aged 14 to 17 years (*n* = 148).

**Table 1:** Influenza vaccination coverage data in the general population by age group (Spain 2018/2019)

	**Vaccine coverage, % (95% CI)**	**Number of individuals investigated**
**Age group**		
< 18 years	6.5 (4.4–8.7)	516
< 5 years	5.2 (0.1–10.3)	74
5–14 years	6.4 (3.6–9.2)	294
14–17 years	7.4 (3.2–11.6)	148
18–64 years	11.1 (9.4–12.2)	3306
18–29 years	4.2 (2.7–5.7)	723
30–55 years	10.8 (9.4–12.2)	1813
56–59 years	15.1 (11.6–18.6)	393
60–64 years	24.6 (20.3–28.9)	379
≥ 65 years	58.0 (56.6–59.5)	4433
65–69 years	40.6 (38.1–43.1)	1498
70–80 years	54.1 (52.2–56.0)	2628
> 80 years	73.5 (68.6–78.4)	307

In adults aged 18 to 64 years (*n* = 3306; 1620 men and 1686 women), 11.1% of those interviewed reported having been vaccinated against influenza: 9.5% of men and 12.7% of women. Coverage data by age subgroups in the population aged between 18 and 64 years are summarized in Table 1.

**Fig. 2:**
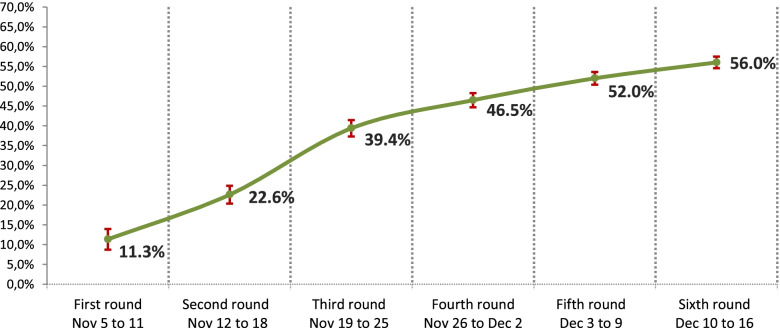
Evolution of the percentages of influenza vaccination coverage in ≥ 65 years by waves (Spain 2018/2019)

Among the individuals analysed aged ≥ 65 years (*n* = 4433), of whom 2021 were men and 2412 women, 56.0% reported having been vaccinated against influenza: 55.9% of men and 66.1% of women. Among individuals aged 65 to 69 years (*n* = 1498), 40.6% had been vaccinated, increasing to 54.1% among adults aged 70 to 80 years (*n* = 2628) and to 73.5% of respondents aged 80 or older (*n* = 307) (Table 1). Figure 2 shows the trend in coverage in adults ≥ 65 years over the 6 waves in the 17 autonomous regions analysed.

Table 2 shows the percentages of vaccination coverage obtained with the Gripometro and the official data provided by the Ministry of Health at the end of the season [15]. In 10 regions, the percentage coverage was above the national average (56.0%), with the highest coverage obtained in Aragon and La Rioja (61% and 64%, respectively).

**Table 2:** Influenza vaccination coverage percentages in the population ≥ 65 years by autonomous region and data origin

Autonomous Region	Percentage coverage	
	**Gripometro data**	**Ministry of Health data**
**Mean**	**56.0%**	**54.2%**
Balearic Islands	44.5%	41.5%
Catalonia	50.5%	51.0%
Murcia	51.3%	52.2%
Valencia region	54.0%	52.0%
Cantabria	54.6%	51.6%
Madrid	54.8%	57.3%
The Canary Islands	55.2%	52.4%
Asturias	56.2%	55.8%
Basque Country	56.5%	58.0%
Galicia	57.2%	58.6%
Extremadura	59.2%	59.6%
Navarre	59.5%	59.8%
Andalusia	60.2%	49.0%
Castile and Leon	60.4%	61.1%
Castilla-La Mancha	60.6%	58.8%
Aragon	61.0%	54.2%
La Rioja	64.3%	64.4%

Comparing the data on vaccination coverage in individuals ≥ 65 years obtained in this study with the official data provided by the Ministry of Health, it was observed that in 15 of the 17 autonomous regions the percentage differences were less than 7% (0.16%-6.74%). More marked differences were observed in only 2 regions: Andalusia, with a difference of 11.8% (60.2% *vs.* 49.0%), and Aragon, with a difference of 6.8% (61.0% *vs.* 54.2%).

#### Analysis of coverage by health status

For the analysis of vaccination coverage in relation to health status, data on chronic patients and pregnant women were pooled in a single group. In this population, which included 2596 cases, 41.7% of those interviewed had already been vaccinated when they answered the questionnaire (Table 3). By age subgroup, among children under 18 years (*n* = 37, small sample base), less than half [16] had not been vaccinated nor did they intend to do so, while among adults aged 18 to 64 years (*n* = 616) these figures were 63.5%, and among adults ≥ 65 years (*n* = 1943), 22.5%.

**Table 3:** Influenza coverage percentages in the general population by health status and age subgroup (Spain 2018/2019)

	**Vaccination coverage,** **% (95% CI)**	**Number of individuals investigated**
**Health status**		
Chronic patients + pregnant women	41.7 (39.8–43.6)	2596
< 18 years	30.1 (15.3–44.9)	37
18–64 years	23.7 (20.4–27.1)	616
≥ 65 years	65.6 (63.5–67.7)	1943
Chronic patients + pregnant women + smokers	30.6 (29.1–32.1)	3451
< 18 years	28.2 (14.3–42.1)	40
18–64 years	15.3 (13.3–17.3)	1234
≥ 65 years	63.9 (61.9–65.9)	2177

Additionally, data on chronic patients, pregnant women and smokers were analysed jointly, with a total of 3451 cases. In this population, 30.6% of those interviewed reported having been vaccinated against influenza. Coverage data in this group by age subgroup are described in Table 3.

### Analysis of coverage in healthcare workers

Of the total of 807 respondents, 39.8% reported having already been vaccinated against influenza. By professional groups, (i) 41.0% of doctors (*n* = 402) and (ii) 38.4% of nurses (*n* = 405) had been vaccinated.

When asked “*Why have you been vaccinated?*”, the main reason among health professionals, in 90.7% of cases, was individual protection, followed by protection of their patients (86.3%) and their family (66.0%). Similarly, when asked “*Why did you decide not to get vaccinated?*”, more than half (54.0%) answered that they did not need to be vaccinated, while about one fifth of respondents answered that the vaccine was not effective (22.6%), that they did not belong to a risk group (18.4%), or that they were already immunized (17.5%) (Table 4).

**Table 4:** Reasons expressed in favor/against of influenza vaccination among healthcare workers (Spain 2018/2019)

Reasons to get vaccinated	Percentage interviewed
Out of habit	15.0%
To avoid infection and to continue working	46.6%
To protect me from the disease	90.7%
To protect my patients from the disease	86.3%
To protect my family from the disease	66.0%
Because I'm in a risk group	11.1%
I’m not in a risk group	18.4%
I don't need to be vaccinated	54.0%
I don’t trust the effectiveness of the vaccine	22.6%
I'm immunized	17.5%

## Discussion

This article describes the Gripometro, a tool created in 2011, which, through a population-based demographic study, allows the levels of influenza vaccine coverage in Spain to be monitored in a periodic way through weekly interviews starting at the beginning of each seasonal influenza vaccination campaign [14]. It also makes it possible to determine the intention to vaccinate in these study groups, as well as the motivations and barriers to vaccination. Data collected using this tool during the 2018–2019 season are also presented, both in the general population and in healthcare workers (primary care doctors and nurses). The results of vaccination coverage obtained by the Gripometro during this season for individuals ≥ 65 years were mostly comparable with the official data presented by the Ministry of Health months later after the end of the vaccination campaign [15], thus demonstrating its usefulness in the periodic monitoring of the percentage vaccination in Spain and, allowing to implement correcting measures or noticing stakeholders if needed.

The results for end-of-season coverage obtained in individuals ≥ 65 years were similar to the data officially published by the Ministry of Health [15]; higher estimates were observed with the Gripometro only in the regions of Andalusia (60.2% *vs.* 49.0%) and Aragon (61.0% *vs.* 54.2%). These differences may be due to differences in the data collection methodology, or in the estimation of the population residing in the regions. In terms of the end-of-season coverage, both the Gripometro data (56.0%) and the official data from the Ministry of Health (54.2%) indicated that only just over half of the investigated individuals were vaccinated against influenza during the study season. According to official data, it appears that the individuals who had not yet been vaccinated, but intended to do so (14.1%), did not eventually get vaccinated. Overall, the average vaccine coverage reached was 10% below the 65% target proposed by the Spanish Ministry of Health for this group, and 20% below the 75% target established by the WHO for this risk group [1, 8]. This suggests that only half of the Spanish population ≥ 65 years benefit from the protection offered by the influenza vaccine, a decisive factor that undoubtedly contributes to the high impact on influenza mortality in this risk group [16].

The coverage recorded in the study in children under 5 years was 5%; these values are to be expected, since in Spain the influenza vaccine is only recommended in children with risk conditions [8]. However, official data show that even in paediatric risk groups, vaccination remains low [17]. In contrast, 22 out of 34 of patients under 18 who were vaccinated did not have chronic conditions nor were they pregnant women, so they did not follow the official vaccination recommendations [8].

Among primary care healthcare workers, both doctors and nurses, the percentage of vaccination coverage was 39.8%, slightly higher than the official data provided by the Ministry of Health for the 2018–19 season, which was 35% including both primary care and hospital healthcare workers [15]. Either way, it is striking that almost half of respondents, 42.7% (44.8% of doctors and 40.2% of nurses), stated that although they had not yet been vaccinated, they intended to do so; however, they did not appear to have done so at all. As this was not a main objective of the study, the statistical power did not allow the estimation of vaccination coverage in healthcare workers, subjects with underlying disease or pregnant women by autonomous region. However, about 20% of the primary healthcare workers were not considered to belong to a risk group or did not trust the effectiveness of the vaccine, as shown in Table 4. In this regard, it seems clear that it is necessary to insist on informing and training healthcare workers about the evidence of the influenza vaccine and its benefits in risk groups, and to strengthen persuasive measures to achieve increased levels of vaccine coverage among healthcare workers and their patients. To this end, the influenza vaccination working group of the Ministry of Health’s *Ponencia de Programa y Registro de Vacunaciones* [Vaccination Programme and Registration Committee] has developed a project to better understand the factors that influence the decision to get vaccinated against influenza in Spain in the 2019–20 season [18]. One of the phases includes a quantitative study of healthcare workers using an online survey sent to all autonomous communities [18]. This group was targeted because of their involvement in both transmitting influenza and recommending vaccination. The results of this survey are not yet known, but are expected to be similar to those obtained by the Gripometro, given its similar methodology. On the other hand, the same working group also performed a qualitative study to the general population, health professionals and risk groups that delves into the most important factors and actors that are influencing behaviours of reluctance towards vaccination. Interestingly, they found that both the general population and professionals showed low awareness of the transmitting role of the disease [19].

Traditional influenza vaccination coverage surveillance systems experience delays due to dependence on reports submitted by medical institutions and on records that are not updated quickly enough. In Spain, the sentinel doctors’ network from each Autonomous Regions provides epidemiological influenza surveillance data to the Ministry of Health at the end of each season. Thus, the knowledge of coverage in real time could enable rapid communication between public health agencies and local governments, facilitating the development of measures to improve coverage. Specifically, monitoring influenza vaccination coverage is important for early detection of anomalies and to propose alternatives. In this sense, the Gripometro is presented as a practical and highly useful tool that may be especially important in times such as the 2020–2021 and successive seasons, in which influenza could coexist with COVID-19. The influenza vaccine is a complementary key weapon, in the context of sanitarian crisis of COVID-19, to prevent a possible collapse of health care. Both the Ministry of Health and the different autonomous regions have stepped up influenza vaccination during 2020–21 season [8]. The data provided by the Gripometro allow close monitoring of influenza vaccine coverage, especially when traditional surveillance systems are mainly focused on COVID-19 epidemics [20].

Like all observational studies, the Gripometro has a number of limitations inherent to the methodology used. For the general population, some response bias may have occurred since it is a telephone survey; for example, people who chose not to get vaccinated may have been less likely to answer the questionnaire. That is an important bias that needs to be addressed. Ensuring participants feel comfortable providing honest answers and emphasizing the anonymity and confidentiality of the surveys may help to mitigate this effect. Furthermore, the fact that calls were made only to landlines may have introduced a selection bias, since the profile of the population that has a landline is different from the one that has only a mobile phone [21]. Specifically, multiple studies have shown that there are differences in sociodemographic and health indicators according to the type of phone available, with worse health indicators among the population who only have a mobile phone [22, 23]. Given that a high percentage of Spanish households use mobile phones (97.4% in 2018 according to data from the Spanish National Institute of Statistics [24]) the results obtained through the Gripometro could be underestimated with respect to the total population resident in Spain, especially for those aged 65 or older. Also, we have to consider that landlines are mostly used by the elderly who have generally a higher vaccination coverage rate. In that sense, coverage data could be overvalued. For future campaigns, the inclusion of mobile phones in the sampling framework should be assessed, in order to avoid selection bias affecting the representativeness of the results obtained. However, one has to consider that since one of the main interest of “The Gripómetro” tool was to get more data in the elderly (≥ 65 years), who has increased risk of complications associated to the flu, fixed quotas were established by autonomous region and by age group (200 questionnaires were assigned in the general population; 50 in the group aged 18 to 64 years and 150 in the group aged 65 to 80 years). Thus, the majority of the individuals investigated were in fact adults ≥ 65 years of age. One the other hand, we have not included people over 80 years old as informants because we consider that they often present difficulties conducting a telephone survey. Thus, we could be missing significant coverage data on this age group that represents an important risk group for vaccination. Ideally, a report of the number of participants not included in the survey, by age group, and the reason for this non-inclusion (not reachable by telephone, refusal to participate, telephone error or others) would help understand the real impact of the selection and response bias in our study. Unfortunately, these data are automatically deleted some months after the completion of the questionnaire.

In summary, the Gripometro is a robust research method that provides real data and trends for influenza vaccine coverage along with other useful information related to vaccination — such as information on attitudes or personal perceptions — in advance of the publication of official data by the Ministry of Health. Real-time tracking of the trend and percentages of vaccine coverage can trigger an almost immediate and continuous response in terms of applying corrective measures where appropriate or reinforcing awareness messages during the vaccination campaign. Additionally, the Gripometro provides coverage data for major population groups that are not available in official reports. In general, the information obtained with this methodology complements the official information provided by the Ministry of Health with regard to the design and planning of annual vaccination campaigns and taking preventive or corrective measures in the different risk groups.

## Supplementary Information

**Additional file 1.** Questionnaires performed to the general population and healthcare professionals used to obtain data for the present study (English translation).

## Data Availability

The datasets used and/or analysed during the current study are available from the corresponding author on reasonable request.
