# Supplementary material for: A tool for early estimation of influenza vaccination coverage in Spanish general population and healthcare workers in the 2018–19 season: the Gripómetro
Source: BMC Public Health. 2022 Apr 25;22:825. doi: 10.1186/s12889-022-13193-x (PMC9036844; doi:10.1186/s12889-022-13193-x)
Supplement: Supplementary file 1 — Additional file 1. Questionnaires performed to the general population and healthcare professionals used to obtain data for the present study (English translation). [file 12889_2022_13193_MOESM1_ESM.docx]

**Supplementary material.**

**S1.** Telephone interview with the general population

**19-310 GRIPÓMETRO 2019 – POPULATION**

**Telephone interview**

Good morning/afternoon, my name is _______ , and I’m an interviewer at AeI Institute of Opinion Studies. We are conducting a study to learn about certain aspects of the flu and its vaccination and we would like your opinion. Would you be so kind as to help us by answering some questions? The estimated duration is around 10 minutes. Your answers will be treated confidentially and grouped with those of other people who collaborate in the study.

Market Research Agencies are required to report adverse events, exposure during pregnancy/breastfeeding, suspected transmission of infectious agents, technical/quality complaints, drug interactions and special situations such as overdose, abuse, misuse, erroneous administration, medication errors, occupational exposure, and lack of effectiveness mentioned during research on any product of the company funding the study. Although whatever you say will be kept confidential, if during the conversation you mention an adverse event (or any of the situations mentioned above) on a specific patient, we must report it to the pharmaceutical company, even if you have already reported it directly to the company or to the Spanish health authorities.

**THANK YOU!**

**C0. To begin with, could you tell me which province you currently live in?** (INTERVIEWER: Do not read).

|  |  | Code |  | Code |
| --- | --- | --- | --- | --- |
|  | Álava | 1 | Jaen | 25 |
|  | Albacete | 2 | León | 26 |
|  | Alicante | 3 | Lleida | 27 |
|  | Almería | 4 | Lugo | 28 |
|  | Asturias | 5 | Madrid | 29 |
|  | Ávila | 6 | Málaga | 30 |
|  | Badajoz | 7 | Murcia | 31 |
|  | Barcelona | 8 | Navarre | 32 |
|  | Burgos | 9 | Orense | 33 |
|  | Cáceres | 10 | Palencia | 34 |
|  | Cadiz | 11 | Pontevedra | 35 |
|  | Cantabria | 12 | La Rioja | 36 |
|  | Castellón | 13 | Salamanca | 37 |
|  | Ciudad Real | 14 | Segovia | 38 |
|  | Cordoba | 15 | Seville | 39 |
|  | La Coruña | 16 | Soria | 40 |
|  | Cuenca | 17 | Tarragona | 41 |
|  | Girona | 18 | Teruel | 42 |
|  | Granada | 19 | Toledo | 43 |
|  | Guadalajara | 20 | Valencia | 44 |
|  | Guipuzcoa | 21 | Valladolid | 45 |
|  | Huelva | 22 | Vizcaya | 46 |
|  | Huesca | 23 | Zamora | 47 |
|  | Balearic Islands | 24 | Zaragoza | 48 |

**C1. What is your nationality?** (INTERVIEWER: Do not read).

Spanish 1

Other nationality 2

**C2. Sex.** (INTERVIEWER: Code directly by voice).

Male 1

Female 2

**C3. How old are you?**

|___|___| Years 99 Don’t know/No answer 🡪 INTERVIEWER: If the respondent does not want to state their exact age, suggest ranges

Less than 18 years 1 🡪 **INTERVIEW INVALID**

18 to 29 years 2

30 to 55 years 3

56 to 59 years 4

60 to 64 years 5

65 to 69 years 6

70 to 80 years 7

More than 80 years 8 🡪 **INTERVIEW INVALID**

Don’t know/No answer 99 🡪 **INTERVIEW INVALID**

**C4.** **Currently, not including yourself, how many people live in your home? Consider both the adults and the children.**

**|__ |___|** people in the home (NOT INCLUDING THE INTERVIEWEE)

**(ONLY IF C4>0** 🡪 **Does not live alone)**

**C5.** **Again, not including yourself, could you please tell me the age and sex of each member of your household, ordering them from youngest to oldest** (CODING: Show as many members as indicated in C4)

|  | **Member 1** | **Member 2** | **Member 3** | **….** | **Member X:** |
| --- | --- | --- | --- | --- | --- |
| A. Sex | Male 1  Female 2 | Male 1  Female 2 | Male 1  Female 2 | Male 1  Female 2 | Male 1  Female 2 |
| B. Age (years) | /_ _ / | /__/ | /__/ | /__/ | /__/ |

**(ASK ALL)**

**MODULE 1: INFLUENZA VACCINE COVERAGE 2019**

**P1. Starting with yourself and then the members of your household, please tell me if you have been vaccinated or if you plan to be vaccinated against the flu during this 2019-2020 campaign?** (INTERVIEWER: Read the options and ask member by member, reading the sex and age. For example: “And the 30-year-old woman?”).

|  | Interviewee:  You | Member 1:  Sex: M/F  Age: /_ _/ | Member 2:  Sex: M/F  Age: /_ _/ | Member 3:  Sex: M/F  Age: /_ _/ | ….  Sex: M/F  Age: /_ _/ | Member X:  Sex: M/F  Age: /_ _/ |
| --- | --- | --- | --- | --- | --- | --- |
| Has already been vaccinated | 1 | 1 | 1 | 1 | 1 | 1 |
| Has not yet been vaccinated but plans to do so | 2 | 2 | 2 | 2 | 2 | 2 |
| Has not been vaccinated and is not going to do so | 3 | 3 | 3 | 3 | 3 | 3 |
| I don't know if they been vaccinated and/or if they plan to do so |  | 99 | 99 | 99 | 99 | 99 |

**P2. Next, I'm going to read a number of problems or circumstances that affect your health. Please tell me if you have any of them.**

**(ONLY IF C4>0** 🡪 **Does not live alone)**

**P2A. And does anyone in your household have any of them? Which?**

(CODING: Rotate items. Ask followed by the individual and by household members)

|  | **P2. Interviewee** | | **P2A. Household members** | | | **(Only if P2A=1 )**  **Which of them?** | | | |
| --- | --- | --- | --- | --- | --- | --- | --- | --- | --- |
|  | Yes | No | Yes | No | DK / NA | Member 1:  Age: /_ _/  Sex: M/F | Member 2:  Age: /_ _/  Sex: M/F | Member 3:  Age: /_ _/  Sex: M/F | ….  Age: /_ _/  Sex: M/F |
| 1. Pregnant **(ONLY IF C2=2 or IF C5A=2 AND C5B>14)** | 1 | 2 | 1 | 2 | 99 |  |  |  |  |
| 2. Heart disease | 1 | 2 | 1 | 2 | 99 |  |  |  |  |
| 3. Respiratory disease (Asthma, Cystic Fibrosis, COPD, etc.) | 1 | 2 | 1 | 2 | 99 |  |  |  |  |
| 4. Cancer | 1 | 2 | 1 | 2 | 99 |  |  |  |  |
| 5. Metabolic diseases such as diabetes | 1 | 2 | 1 | 2 | 99 |  |  |  |  |
| 6. Liver problems | 1 | 2 | 1 | 2 | 99 |  |  |  |  |
| 7. Kidney problems | 1 | 2 | 1 | 2 | 99 |  |  |  |  |
| 8. Diagnosed anaemia | 1 | 2 | 1 | 2 | 99 |  |  |  |  |
| 9. Diagnosed morbid obesity | 1 | 2 | 1 | 2 | 99 |  |  |  |  |
| 10. Immunosuppression (due to disease or treatment). | 1 | 2 | 1 | 2 | 99 |  |  |  |  |
| 11. Chronic neurological diseases such as cerebral palsy or intellectual disability, muscular dystrophy, etc.) | 1 | 2 | 1 | 2 | 99 |  |  |  |  |
| 12. Disability involving mobility problems | 1 | 2 | 1 | 2 | 99 |  |  |  |  |
| 13. Smokes **(Only if C5B> 14 )** | 1 | 2 | 1 | 2 | 99 |  |  |  |  |

**From now on, all the questions I am going to ask you are just about you.**

**MODULE 2: INFLUENZA VACCINE-RELATED 2019-2020**

**(ONLY IF THEY HAVE BEEN OR INTEND TO GET VACCINATED – P1.1=1 OR 2)**

**P3. The decision to get vaccinated against the flu this year**… (INTERVIEWER: Single answer).

You took the decision 1

You decided because was recommended to you 2

**(ONLY IF RECOMMENDED - P3=2)**

**P3A. And who recommended it?** (INTERVIEWER: Do not read. Multiple answer)

|  | Yes | No |
| --- | --- | --- |
| Healthcare professional (doctor or nurse) | 1 | 2 |
| Pharmacist | 1 | 2 |
| Acquaintance or relative | 1 | 2 |
| Staff at the company where I work | 1 | 2 |
| Other (Specify:___________________________________) | 1 | 2 |

**(IF ALREADY VACCINATED – P1.1=1)**

**P4A. Where did you receive the vaccine?** (INTERVIEWER: Do not read. Single answer).

**(IF THEY INTEND TO BUT HAVE NOT YET DONE SO – P1.1=2)**

**P4B. You’ve told me that you plan to get vaccinated this year. Where do you plan to do so?** (INTERVIEWER: Do not read. Single answer).

In the company where they work 1

At home 2

In a public hospital 3

In a public health centre 4

In a private centre 5

Other (Specify:___________________________) 97

**(IF ALREADY VACCINATED – P1.1=1)**

**P5. Did you or someone close to you go to the pharmacy to buy the vaccine?** (INTERVIEWER: Do not read. Single answer).

Yes 1

No 2

**MODULE 3: EXPOSURE AND PRESCRIPTION OF THE CURRENT INFLUENZA CAMPAIGN**

**(TO ALL)**

**P6. Have you gone to the health centre to receive any kind of health care from October until now?** (INTERVIEWER: Do not read. Single answer. CODING: If P4A=4 or P4A=5, Do not give the option to say “no” because we know they have gone to the medical centre, at least to get vaccinated).

Yes 1

No 2

Yes, but I only went to get the flu vaccine……………………………..…………….....3

**(ONLY IF P6 = 1 - Has gone to the primary care physician)**

**P7A. And at this visit to the health centre, if you have gone more than once, please focus on the last time…The Primary Care Physician…?**

**P7B. And the nurse at your health centre?**

(INTERVIEWER: Read options. Single answer).

|  | Urged them to get vaccinated | Recommended vaccination but without insisting | Did not mention the vaccine | Said it was better not to get vaccinated | Had no contact with them |
| --- | --- | --- | --- | --- | --- |
| P10A. Doctor | 1 | 2 | 3 | 4 | 5 |
| P10B. Nurse | 1 | 2 | 3 | 4 | 5 |

**MODULE 4: EXPERIENCE WITH THE INFLUENZA VACCINE**

**Now we'd like to know what your experience with the flu vaccine has been in the past.**

**P8. Have you had the flu vaccination in the last 3 years? To answer, do not take into account what you have done in the current vaccination campaign (2019-2020).** (INTERVIEWER: Do not read. Single answer).

Yes 1

No 2

**PROFILE DEFINITION BASED ON THEIR RELATIONSHIP WITH THE INFLUENZA VACCINE:**

| - **If vaccinated in the past (P8=1) but won't do so this year (P1.1=3)** | LOST |
| --- | --- |
| - **If not vaccinated in the past (P8=2) and will not do so this year either (P1.1=3)** | NOT CONVINCED |
| - **If not vaccinated in the past (P8=2) but has done or will do so this year (P1.1=1/2)** | WON |
| - **If ever vaccinated in the past (P8=1) and has done or will do so this year (P1.1=1/2)** | FAITHFUL |

**PROFILE=LOST (IF P8=1 AND P1.1=3)**

**P9. You’ve told me that on other occasions you've been vaccinated against the flu, but that you’re not going to do so this year. What were the main reasons for this decision?** (INTERVIEWER: Do not read. Spontaneous and multiple answer. CODING: Rotate)

|  | Yes | No |
| --- | --- | --- |
| They had a bad experience with the influenza vaccine | 1 | 2 |
| They have lost confidence that the vaccine will protect them from the flu | 1 | 2 |
| They are afraid of needles | 1 | 2 |
| They have a disease and cannot get the vaccine | 1 | 2 |
| The bother of going to the health centre and getting it | 1 | 2 |
| Other reason not mentioned: Specify:___________________ | 1 | 2 |

**PROFILE= NOT CONVINCED (IF P8=2 AND P1.1=3)**

**P10. You’ve told me that you haven’t been vaccinated in the last 3 years and that you don’t plan to do so this year either. What are the main reasons for your decision?** (INTERVIEWER: Do not read. Spontaneous answer CODING: Rotate)

|  | Yes | No |
| --- | --- | --- |
| They have no disease that makes them prone to influenza **ONLY IF P2 DOES NOT INDICATE ANY DISEASE)** | 1 | 2 |
| Influenza is a bearable disease | 1 | 2 |
| There’s not that much risk of infection | 1 | 2 |
| They are against vaccines | 1 | 2 |
| They don’t trust the vaccine to protect them from the flu | 1 | 2 |
| Bad experiences with the vaccine in their environment | 1 | 2 |
| They are afraid of needles | 1 | 2 |
| The bother of going to the health centre and getting it | 1 | 2 |
| Because I'm generally in good health | 1 | 2 |
| Other reason not mentioned: Specify:___________________ | 1 | 2 |

**PROFILE=LOST (IF P8=2 AND P1.1= 1/2)**

**(IF ALREADY VACCINATED – P1.1=1)**

**P11. You’ve told me that you haven’t been vaccinated against the flu in the last 3 years, but that you have this year. Could you tell me why?** (INTERVIEWER: Do not read. Spontaneous and multiple answer. CODING: Rotate).

**(IF THEY INTEND TO BUT HAVE NOT YET DONE SO – P1.1=2)**

**P11. You’ve told me that you haven’t been vaccinated against the flu in the last 3 years, but that you intend to do so this year. What were the main reasons for this decision?** (INTERVIEWER: Do not read. Spontaneous answer. Multiple answer. CODING: Rotate)

|  | Yes | No |
| --- | --- | --- |
| There are pregnant women at home or among their friends or they are pregnant | 1 | 2 |
| They have a disease that puts them in a risk group for influenza **(ONLY IF ANY INDICATED IN P2)** | 1 | 2 |
| Because they’re not generally in good health | 1 | 2 |
| They or someone close to them had a bad experience due to the severity of the flu | 1 | 2 |
| Because they are at risk of catching the flu due to their profession | 1 | 2 |
| Because they don’t like missing work or their obligations due to the flu | 1 | 2 |
| Because they don’t want to spread the flu around them | 1 | 2 |
| Because it is recommended by the health authorities | 1 | 2 |
| At the insistence of the doctor or nurse at their health centre or workplace | 1 | 2 |
| Other (Specify:________________________________________________________) | 1 | 2 |

**PROFILE=FAITHFUL (IF P8=2 AND P1.1= 1/2)**

**(IF ALREADY VACCINATED – P1.1=1)**

**P12. You’ve told me that in previous years, and also this time, you’ve been vaccinated against the flu. What are the main reasons for your decision?** (INTERVIEWER: Do not read. Spontaneous answer. Multiple answer. CODING: Rotate).

**(IF THEY INTEND TO BUT HAVE NOT YET DONE SO – P1.1=2)**

**P13. You’ve told me that in the past you’ve been vaccinated against the flu and that this year you also intend to do so. What are the main reasons for your decision?** (INTERVIEWER: Do not read. Spontaneous answer. Multiple answer. CODING: Rotate).

|  | Yes | No |
| --- | --- | --- |
| There are pregnant women at home or among their friends or they are pregnant | 1 | 2 |
| They have a disease that puts them in a risk group for influenza **(ONLY IF ANY INDICATED IN P2)** | 1 | 2 |
| Because they’re not generally in good health | 1 | 2 |
| They or someone close to them had a bad experience due to the severity of the flu | 1 | 2 |
| Because they are at risk of catching the flu due to their profession | 1 | 2 |
| Because they don’t like missing work or their obligations due to the flu | 1 | 2 |
| Because they don’t want to spread the flu around them | 1 | 2 |
| Because it is recommended by the health authorities | 1 | 2 |
| At the insistence of the doctor or nurse at their health centre or workplace | 1 | 2 |
| Other (Specify:________________________________________________________) | 1 | 2 |

**MODULE 5: EXPERIENCE AND PERCEPTION OF INFLUENZA**

**(ASK ALL)**

**To conclude, we would like to know now what your recent experience with the flu has been.**

**P14. Have you caught the flu in the last 3 years?** (INTERVIEWER: Do not read. Single answer).

Yes 1

No 2

**(ONLY IF P14=1 – Has caught the flu in the last 3 years)**

**P15. Referring to the last time you had the flu, how severe was it?** (INTERVIEWER: Read. Single answer).

Mild 1

Moderate 2

They had to go to the emergency service at the surgery/health centre 3

They had to go to the hospital emergency department 4

They were admitted to hospital 5

**P16. Continuing with the latest flu, did it force you to take sick leave or miss work?** (INTERVIEWER: Do not read. Single answer).

Yes 1

No 2

I was not working 3

**P17. And to what extent did it force you to slow down your pace of work, that is, to stop doing your daily activities?** (INTERVIEWER: Read. Single answer).

A lot 4

Quite a lot 3

Very little 2

Not at all 1

**(TO ALL)**

**P18. In your opinion, and thinking about your own health, the flu is…** (INTERVIEWER: Single answer).

A troublesome but tolerable disease 1

A troublesome disease that can cause complications 2

A disease that can be serious 3

A disease that can cause death 4

**CLASSIFICATION DATA**

**Finally, I’m going to ask you some personal information to be able to classify your answers.**

**D.1 What is your employment situation?** (INTERVIEWER: Read. Single answer)

I work outside the home 1

Unemployed 2

Retired 3

Student 4

Housewife 5

Other (Specify:______) 97

**(ONLY IF D1=1 )**

**D2. Please tell me which of the following best matches the type of work you do:**

(INTERVIEWER: Read. Single answer)

I work directly with the public (public counters, registry offices, shops, etc.) .........................1

I work with risk groups (children, the elderly, etc.) but I’m not a healthcare worker...............2

I work as a healthcare professional (doctors/nurses)...............................................................3

None of the above.....................................................................................................................6

**(ASK ALL)**

**D3. What level of education have you completed?** (INTERVIEWER: Do not read. Single answer)

He/she did not complete primary education (school) 1

Primary education (school) 2

Secondary education (high school/modules) 3

University education 4

**D4. Do any of your family or people close to you work in the healthcare field?** (INTERVIEWER: Do not read. Single answer)

Yes 1

No 2

**D5. Do you currently have private health insurance?** (INTERVIEWER: Do not read. Single answer)

Yes 1

No 2

**D6. You would say your health is…**(INTERVIEWER: Read. Single answer)

Very good 1

Good 2

Average.................................. 3

Poor 4

Very poor 5

**D8. To what extent would you say that you use public transport in your daily commute? To answer, consider both urban and intercity.** (INTERVIEWER: Read. Single answer)

A lot 4

Quite a lot 3

Very little 2

Not at all 1

**Thank you very much**

**S2. Healthcare professionals’ online interview**

**19-310 GRIPÓMETRO 2019 – HEALTHCARE PROFESSIONALS**

**ONLINE interview**

As you were informed a few days ago, at the AeI, an Institute of Opinion Studies, we are conducting a study among health professionals to learn about certain aspects related to influenza and its vaccination, and we would like your opinion.

The estimated duration is less than 10 minutes. Your answers will be treated confidentially and grouped with those of other people who collaborate in the study.

Market Research Agencies are required to report adverse events, exposure during pregnancy/breastfeeding exposure, suspected transmission of infectious agents, technical/quality complaints, drug interactions and special situations such as overdose, abuse, misuse, erroneous administration, medication errors, occupational exposure, and lack of effectiveness mentioned during research on any product of the company funding the study. Although whatever you say will be kept confidential, if during the conversation you mention an adverse event (or any of the situations mentioned above) on a specific patient, we must report it to the pharmaceutical company, even if you have already reported it directly to the company or to the Spanish health authorities.

**THANK YOU!**

**PROFILE DEFINITION**

**C0. Please indicate the type of centre where you currently work**

Health Centre -Specialities 1

Hospital 2🡪 **INTERVIEW INVALID**

**C1. You are a...**

Doctor 1

Nurse 2

Other 97🡪 **INTERVIEW INVALID**

**C2. What is your specialty?**

Primary Care 1

Other 97🡪 **INTERVIEW INVALID**

**C3. And within primary care, your patients are...?**

Adults only 1

Adults and children 2

Children only……………………………………3 🡪 **INTERVIEW INVALID**

**C4. Type of centre where you work:**

Public 1

Public and Private ……………………………2

Private 3🡪 **INTERVIEW INVALID**

(If C4=2, ‘From now on, we will refer exclusively to your professional experience in the public sphere’ appears in the formulation)

**C5. Region where the centre is located**

| 1 | Araba/Álava | 26 | La Rioja |
| --- | --- | --- | --- |
| 2 | Albacete | 27 | Lugo |
| 3 | Alicante | 28 | Madrid |
| 4 | Almería | 29 | Málaga |
| 5 | Ávila | 30 | Murcia |
| 6 | Badajoz | 31 | Navarre |
| 7 | Balearic Islands | 32 | Ourense |
| 8 | Barcelona | 33 | Asturias |
| 9 | Burgos | 34 | Palencia |
| 10 | Cáceres | 35 | Las Palmas |
| 11 | Cadiz | 36 | Pontevedra |
| 12 | Castellón | 37 | Salamanca |
| 13 | Ciudad Real | 38 | Santa Cruz de Tenerife |
| 14 | Cordoba | 39 | Cantabria |
| 15 | La Coruña | 40 | Segovia |
| 16 | Cuenca | 41 | Seville |
| 17 | Girona | 42 | Soria |
| 18 | Granada | 43 | Tarragona |
| 19 | Guadalajara | 44 | Teruel |
| 20 | Guipuzcoa | 45 | Toledo |
| 21 | Huelva | 46 | Valencia |
| 22 | Huesca | 47 | Valladolid |
| 23 | Jaen | 48 | Bizkaia |
| 24 | León | 49 | Zamora |
| 25 | Lleida | 50 | Zaragoza |

**C6.** **Currently, not including yourself, how many people live in your home? Consider both the adults and the children.**

**|__ |___|** people in the home (NOT INCLUDING THE INTERVIEWEE)

**(ONLY IF C4>0** 🡪 **Does not live alone)**

**C7.** **Again, not including yourself, could you please indicate the age and sex of each member of your household, ordering them from youngest to oldest** (CODING: Show as many members as indicated in C4)

|  | **Member 1** | **Member 2** | **Member 3** | **….** | **Member X:** |
| --- | --- | --- | --- | --- | --- |
| A. Sex | Male 1  Female 2 | Male 1  Female 2 | Male 1  Female 2 | Male 1  Female 2 | Male 1  Female 2 |
| B. Age (years) | /_ _ / | /__/ | /__/ | /__/ | /__/ |

**(ASK ALL)**

**MODULE 1: INFLUENZA VACCINE COVERAGE 2019**

**C8. Starting with yourself and then the members of your household, please tell me if you have been vaccinated or if you plan to get vaccinated against influenza during this 2019-2020 campaign?** (INTERVIEWER: Read the options and ask member by member, reading the sex and age. For example: “And the 30-year-old woman?”).

|  | Interviewee:  You | Member 1:  Sex: M/F  Age: /_ _/ | Member 2:  Sex: M/F  Age: /_ _/ | Member 3:  Sex: M/F  Age: /_ _/ | ….  Sex: M/F  Age: /_ _/ | Member X:  Sex: M/F  Age: /_ _/ |
| --- | --- | --- | --- | --- | --- | --- |
| Has already been vaccinated | 1 | 1 | 1 | 1 | 1 | 1 |
| Has not yet been vaccinated but plans to do so | 2 | 2 | 2 | 2 | 2 | 2 |
| Has not been vaccinated and is not going to do so | 3 | 3 | 3 | 3 | 3 | 3 |
| I don't know if they been vaccinated and/or if they plan to do so |  | 99 | 99 | 99 | 99 | 99 |

**STARTING POINT: INFLUENZA PREVENTION**

**We would now like to hear your opinion on the need to prevent influenza.**

**P1. For the following defined profiles, based on your professional and/or personal activity, please indicate to what extent you think it may be advisable to get the influenza vaccine. To answer, use the scale: Essential, highly advisable, advisable, optional or not recommended.** (CODING: Rotate items. Single answer for each group).

|  | Essential | Highly Advisable | Advisable | Optional | Not recommended |
| --- | --- | --- | --- | --- | --- |
| 1. People who work with the elderly or children or who are in contact with vulnerable people | 5 | 4 | 3 | 2 | 1 |
| 2. Doctors | 5 | 4 | 3 | 2 | 1 |
| 3. Nurses | 5 | 4 | 3 | 2 | 1 |
| 4. Other staff (neither doctors nor nurses) who work in hospitals or health centres and who have direct patient contact. | 5 | 4 | 3 | 2 | 1 |
| 5. Workers who have to deal directly with the public (public counters, registry offices, shops, etc .) | 5 | 4 | 3 | 2 | 1 |
| 6. Workers directly exposed to poultry or swine on farms. | 5 | 4 | 3 | 2 | 1 |
| 7. Essential public service workers (fire, police, civil defence, emergency services, etc.) | 5 | 4 | 3 | 2 | 1 |
| 8. People who live at home with children, elderly people or people with poor health | 5 | 4 | 3 | 2 | 1 |

**P.2. Now, for the following risk groups, tell me to what extent do you think influenza can become: a troublesome but uncomplicated disease, a troublesome disease that can cause complications, that can become serious, or that can lead to death?** (CODING: Rotate items. Single answer for each group).

|  | **Influenza is…** | | | |  |
| --- | --- | --- | --- | --- | --- |
|  | A troublesome but uncomplicated disease | A troublesome disease that can cause complications | A disease that can become serious | A disease that can cause death | |
| 1. Children under 5 years old | 1 | 2 | 3 | 4 | |
| 2. Over 65 years old | 1 | 2 | 3 | 4 | |
| 3. Pregnant women | 1 | 2 | 3 | 4 | |
| 4. People with heart disease | 1 | 2 | 3 | 4 | |
| 5. People with lung diseases (emphysema, chronic bronchitis, asthma, etc.) | 1 | 2 | 3 | 4 | |
| 6. People with cancer | 1 | 2 | 3 | 4 | |
| 7. People with metabolic diseases (including DM) | 1 | 2 | 3 | 4 | |
| 8. People with chronic liver disease | 1 | 2 | 3 | 4 | |
| 9. People with haemoglobinopathies and anaemias | 1 | 2 | 3 | 4 | |
| 10. People with kidney failure | 1 | 2 | 3 | 4 | |
| 11. People with morbid obesity | 1 | 2 | 3 | 4 | |
| 12. People with immunosuppression, whether caused by disease (e.g. HIV) or by drugs | 1 | 2 | 3 | 4 | |
| 13. People with chronic neurological diseases (examples: cerebral palsy or intellectual disability, muscular dystrophy, etc.) | 1 | 2 | 3 | 4 | |
| 14. People with a disability that involves mobility problems |  |  |  |  | |
| 15. Smokers | 1 | 2 | 3 | 4 | |

**P3. And what do you think influenza might be like for people who do not belong to any of the risk groups defined in the previous question? A troublesome but uncomplicated disease, a troublesome disease that can cause complications, that can become serious, or that can lead to death.**

|  | **Influenza is…** | | | |  |
| --- | --- | --- | --- | --- | --- |
|  | A troublesome but uncomplicated disease | A troublesome disease that can cause complications | A disease that can become serious | A disease that can cause death | |
| People who are not in a risk group | 1 | 2 | 3 | 4 | |

**INFLUENZA VACCINATION CAMPAIGN: PATIENT PERSPECTIVE**

**Now I'm going to ask you some questions about the current influenza vaccination campaign, focusing on your patients.**

**P4. During this campaign, what are you saying to patients who come to your health centre about influenza vaccination?** (CODING: Single answer for each profile).

|  | I don’t say anything | I inform them that they can get vaccinated but I don’t insist | I recommend or advise them to get vaccinated | I urge them to get vaccinated |
| --- | --- | --- | --- | --- |
| To a patient **under 65** who is in a **risk group** | 1 | 2 | 3 | 4 |
| To a patient **under 65** in **normal health** | 1 | 2 | 3 | 4 |
| To a patient **aged 65 or older** who is in a **risk group** | 1 | 2 | 3 | 4 |
| To a patient **aged 65 years or older** in **normal health** | 1 | 2 | 3 | 4 |

**(ONLY IF C3=2** 🡪 **Your patients include children)**

**P4A. You said that your patients also include children. In line with this, what are you saying to parents and/or guardians of children about influenza vaccination?** (CODING: Single answer for each profile).

|  | I don’t say anything | I inform them that they can vaccinate their child but without insisting | I recommend or advise them to vaccinate their child | I urge them to vaccinate their child |
| --- | --- | --- | --- | --- |
| To a **child under 2 years** in **normal health** | 1 | 2 | 3 | 4 |
| To a child **aged 2 to 5** in **normal health** | 1 | 2 | 3 | 4 |
| To a child **aged 5 to 14** in **normal health** | 1 | 2 | 3 | 4 |

**(ASK ALL)**

**P5. And with regard to the previous campaign, in general, how strongly are you recommending vaccination?** (CODING: Single answer)

More strongly than in the previous campaign 4

Just as strongly 3

Less strongly 2

I'm not recommending vaccination 1

**(ONLY IF P5=4** 🡪 **Recommends more strongly)**

**P6A. Why did you recommend vaccination more strongly this year? Please write in detail everything that has made you recommend it to a greater extent.**

**(ONLY IF P5=2** 🡪 **Recommends less strongly)**

**P6B. Why did you recommend vaccination less strongly this year? Please write in detail everything that has made you recommend it to a lesser extent.**

**(ASK ALL)**

**P7. Continuing with the current influenza campaign, please indicate if you have observed any of the following activities or appeals for patients to be vaccinated at your workplace.** (CODING: Multiple answers)

|  | Yes | No |
| --- | --- | --- |
| Patient leaflets | 1 | 2 |
| Patient posters | 1 | 2 |
| Patient in-person meetings | 1 | 2 |
| Sending SMS reminders to patients | 1 | 2 |
| Other type of activity 1:_________________ | 1 | 2 |
| Other type of activity 2:_________________ | 1 | 2 |
| I have not observed any activities or appeals | 1 | 2 |

**P8. In your experience, on average, how many days sick leave do patients have due to seasonal influenza?**

|___|___| sick days for influenza in patients in normal health. 999. DK/NA

|___|___| sick days for influenza in patients in a risk group 999. DK/NA

**INFLUENZA VACCINATION CAMPAIGN: PROFESSIONAL PERSPECTIVE**

**While earlier we talked about the influenza campaign among your patients, we're now going to focus on healthcare professionals, starting with you.**

**P9. Do you have any chronic health problems or illnesses?** (CODING: Single answer).

Yes 1

No 2

**(ONLY IF P9=1** 🡪 **Has a chronic illness)**

**P10. Specifically, what chronic illness do you have?** (CODING: Rotate items. Multiple answer)

|  | Yes | No |
| --- | --- | --- |
| Heart disease | 1 | 2 |
| Respiratory disease (emphysema, chronic bronchitis, asthma, etc.) | 1 | 2 |
| Cancer | 1 | 2 |
| Metabolic disease (including Diabetes Mellitus) | 1 | 2 |
| Chronic liver disease | 1 | 2 |
| Haemoglobinopathies and/anaemias | 1 | 2 |
| Chronic renal failure | 1 | 2 |
| Morbid obesity | 1 | 2 |
| Disease of the immune system | 1 | 2 |
| Other (specify:______________________________________) | 1 | 2 |

**(TO ALL)**

**P10A. Are you a smoker?** (CODING: Single answer).

Yes 1

No 2

**P11. And have you been vaccinated against influenza in this campaign (2019-2020)?** (CODING: Single answer).

Yes, I have already done so 1

I have not done so yet, but I intend to 2

I have not been vaccinated nor do I intend to do so

**[ONLY IF P11=1** 🡪 **HAS ALREADY BEEN VACCINATED]**

**P22A. And were you vaccinated in your workplace or at another facility?**

In my workplace 1

At another facility 2

**P22B. And could you tell me what date you had the vaccine?**

**_____________________________________________________________________**

**[ONLY IF P11=2** 🡪 **INTENDS TO DO SO]**

**P23A. And will you get the influenza vaccine in your workplace or at another facility?**

In my workplace 1

At another facility 2

**P23B. And when do you plan to do that?**

I’ve already made the appointment 1

I will do so within the next 15 days 2

I'm still not clear when I'll get the vaccine 3

**(ONLY IF P11=1 or P11=2** 🡪 **If you have been vaccinated or you intend to do so)**

**P12A. And what are the main reasons you have chosen to get vaccinated?** (CODING: Rotate items. Multiple answer).

|  | Yes | No |
| --- | --- | --- |
| To protect myself from the disease | 1 | 2 |
| To protect my patients from the disease | 1 | 2 |
| To protect my family from the disease | 1 | 2 |
| To avoid infection and to continue working | 1 | 2 |
| Out of habit | 1 | 2 |
| Because I’m in a risk group (ONLY IF P9=1) | 1 | 2 |
| Other reason:________________________ | 1 | 2 |

**(ONLY IF P11=3** 🡪 **Has not and will not be vaccinated)**

**P12B. And what are the main reasons why you have chosen not to get vaccinated?** (CODING: Multiple answer).

|  | Yes | No |
| --- | --- | --- |
| I don't need to be vaccinated, I'm in good health | 1 | 2 |
| I'm already immunized because of my exposure to disease | 1 | 2 |
| I’m not in a risk group (ONLY IF P9=2) | 1 | 2 |
| I don’t think it’s necessary | 1 | 2 |
| I've done it other years and even so, I've got sick from the flu | 1 | 2 |
| Other reason:________________________ | 1 | 2 |

**(TO ALL)**

**P13. Continuing with the current influenza campaign, please indicate if you have observed any of the following activities or appeals to get vaccinated as a healthcare professional at your workplace?** (CODING: Multiple answer)

|  | Yes | No |
| --- | --- | --- |
| Leaflets for professionals | 1 | 2 |
| Posters for professionals | 1 | 2 |
| In-person meetings for professionals | 1 | 2 |
| Letters or newsletters from the Ministry of Public Health informing staff about the campaign | 1 | 2 |
| Health centre prevention department | 1 | 2 |
| Other type of activity 1:_________________ | 1 | 2 |
| Other type of activity 2:_________________ | 1 | 2 |
| No activity or appeal | 1 | 2 |

**(ONLY IF P13=1** 🡪 **Only if you have observed any of the actions)**

**P14. To what extent do you think these materials or actions influence your assessment of influenza vaccination for yourself?** (CODING: Single answer)

A lot 4

Quite a lot 3

Very little 2

Not at all 1

**(ASK ALL)**

**P15. What is your Health Centre's policy regarding influenza vaccination for its staff?** (CODING: Single answer).

They inform us about vaccination 1

They recommend vaccination, and each of us decides what to do 2

They insist that we get vaccinated, both those who want to and those who don't 3

**P16.** **Based on your perception or knowledge, what percentage of healthcare professionals do you think are vaccinated against influenza?**

|___|__|___| % of healthcare professionals who get vaccinated

**P17. And specifically in your health centre/work, what percentage of healthcare professionals do you think you are vaccinated against influenza?**

|___|__|___| % of healthcare professionals vaccinated in your health centre

**P18. In your opinion, how important do you think influenza prevention and vaccination are among healthcare professionals?** (CODING: Single answer).

Prevention and vaccination are very important 4

Prevention and vaccination are quite important 3

Prevention and vaccination are not very important 2

Prevention and vaccination are not important at all 1

**(ONLY IF P18=4 or P18=3** 🡪 **They consider it to be very or quite important)**

**P19A. Why do you think influenza prevention and vaccination is important among healthcare workers? Please explain your answer in detail.**

**(ONLY IF P18=2 or P18=1** 🡪 **They consider it to be not very important or not important at all)**

**P19B. Why do you think that influenza prevention and vaccination is NOT important among healthcare workers? Please explain your answer in detail.**

**(TO ALL)**

**P20. What do you think might motivate your colleagues to get vaccinated against seasonal flu?** (CODING: Rotate items. Multiple answer).

|  | Yes | No |
| --- | --- | --- |
| Greater communication from the Ministry / Public Health | 1 | 2 |
| Seeing other colleagues vaccinated | 1 | 2 |
| More information about the effectiveness of the vaccine | 1 | 2 |
| That within the Centre, campaigns were conducted aimed at us as a target audience informing us about the campaign | 1 | 2 |
| Raising awareness of the importance of vaccinating healthcare professionals so that neither they nor the patient become infected | 1 | 2 |
| Having greater knowledge of the burden caused by influenza in Spain: deaths, visits to PC, visits to the emergency department, etc. | 1 | 2 |
| Other (specify:______________________________________) | 1 | 2 |
| Not at all | 1 | 2 |

**P21. Could you tell me roughly what influenza vaccination coverage has been among healthcare professionals in recent seasons?**

|___|___|___| % vaccination coverage among healthcare professionals. 999. DK/NA

**P24. Could you tell me if your autonomous region's influenza vaccine for this 2019-2020 campaign is 3 or 4 strains?**

3 strains 1

4 strains 2

I don't know 99

**CLASSIFICATION DATA**

**Finally, I would be grateful if you could answer a few questions so that we can classify your answers.**

**D1. In the health centre where your work, including yourself…**

A. Approximately…How many nurses are there? /_ _ / 999.DK/NA

B. Approximately…How many doctors are there? /_ _/ 999.DK/NA

**D2. In which area is the centre where you work?**

Centre of a medium or large city 1

Suburbs of a medium or large city 2

Centre of a small town 3

Suburbs of a small town 4

Rural area 5

**(ASK ALL)**

**D3. AGE:**

**|___|___| years**

**D4. SEX:**

Male 1

Female 2

**(ONLY IF D4=2** 🡪 **FEMALE)**

**D4A. Are you pregnant?**

Yes ..........................................1

No 2
